# Correction: Predicting drug response from single-cell expression profiles of tumours

**DOI:** 10.1186/s12916-024-03289-z

**Published:** 2024-02-16

**Authors:** Simona Pellecchia, Gaetano Viscido, Melania Franchini, Gennaro Gambardella

**Affiliations:** 1 Telethon Institute of Genetics and Medicine, Naples, Italy; 2 Genomics and Experimental Medicine Program, Scuola Superiore Meridionale, Naples, Italy; 3 Department of Chemical, Materials and Industrial Engineering, University of Naples Federico II, Naples, Italy; 4 Department of Electrical Engineering and Information Technology, University of Naples Federico II, Naples, Italy

**Correction** **:** **BMC Med 21, 476 (2023)**

**https://doi.org/10.1186/s12916-023-03182-1**

Upon reviewing the published manuscript [1], the authors identified a errors affecting Figure 2 that require clarification:

1. In Figure 2C, the wild-type (WT) and mutated TP53 cell lines were incorrectly colour-coded.

2. In Figure 2D and 2E, the labels for TP53 mutated cells and wild type were inadvertently inverted.

3. There is a typographical error in the caption of Fig. 2, where the word "XX" should be replaced with "24".

Figure 2
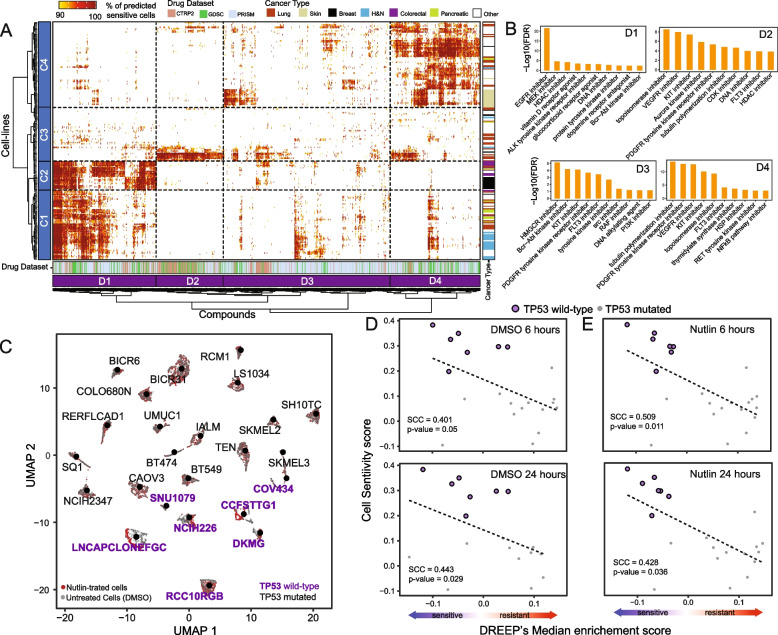


The corrected version of Figure 2 and its caption can be viewed ahead for reference.

In addition, the following line in the main text should be changed from:

"As depicted in Fig. 2C, seven of the 24 cell lines within this dataset harbour a missense mutation in the TP53 gene, while all other cell lines retain the wild-type (WT) TP53 form."

to:

"As depicted in Figure 2, seven of the 24 cell lines within this dataset retain the wild-type (WT) TP53 form, while all other cell lines harbour a missense mutation in the TP53 gene."
