# Supplementary material for: Predicting drug response from single-cell expression profiles of tumours
Source: BMC Med. 2023 Dec 1;21:476. doi: 10.1186/s12916-023-03182-1 (PMC10693176; doi:10.1186/s12916-023-03182-1)
Supplement: Supplementary file 1 — Additional file 1: Figure S1. GPDS consistency across viability datasets. Figure S2. DREEP’s ROC AUC distribution across cancer types. Figure S3. DREEP Method Performance on Pan-Cancer Dataset using IC50-Derived GPDS. Figure S4. Drug prediction performance using absolute expression to extract relevant genes of a cell. Figure S5. Performance comparison between DREEP and other single cell drug prediction tool. Figure S6. DREEP's predictions for McFarland et al. Dataset. [file 12916_2023_3182_MOESM1_ESM.pdf]

Predicting drug response from single-cell expression profiles of tumours

AUTHORS

Simona Pellecchia[1,4,†], Gaetano Viscido[1,2, †], Melania Franchini[1,3], Gennaro Gambardella[1,#]

AFFILIATIONS

[1]Telethon Institute of Genetics and Medicine, Naples, Italy
[2]University of Naples Federico II, Department of Chemical, Materials and Industrial Engineering, Naples, Italy
[3]University of Naples Federico II, Department of Electrical Engineering and Information Technology, Naples, Italy
[4]Scuola Superiore Meridionale, Genomics and Experimental Medicine Program, Naples, Italy

[†]These authors contributed equally to the work.

[#]CORRESPONDENCE: gambardella@tigem.it

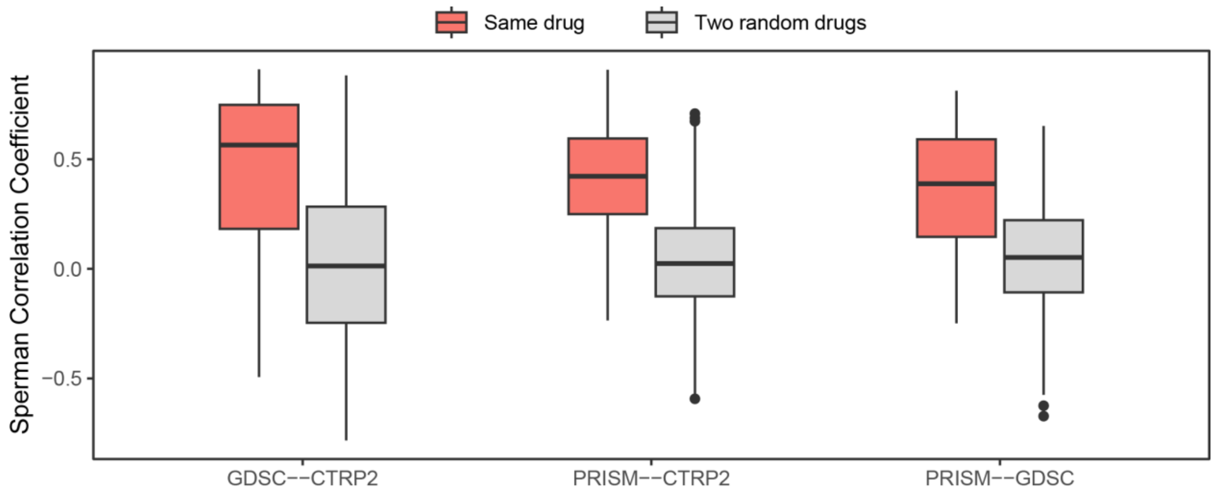

Figure S1 – GPDS consistency across viability datasets. This figure illustrates the Spearman Correlation Coefficient (SCC) distribution between two GDPS representing the same drug but derived from distinct viability datasets (depicted in the red boxplot). For comparison, the SCC distribution between two randomly selected drugs is also shown (grey boxplot).

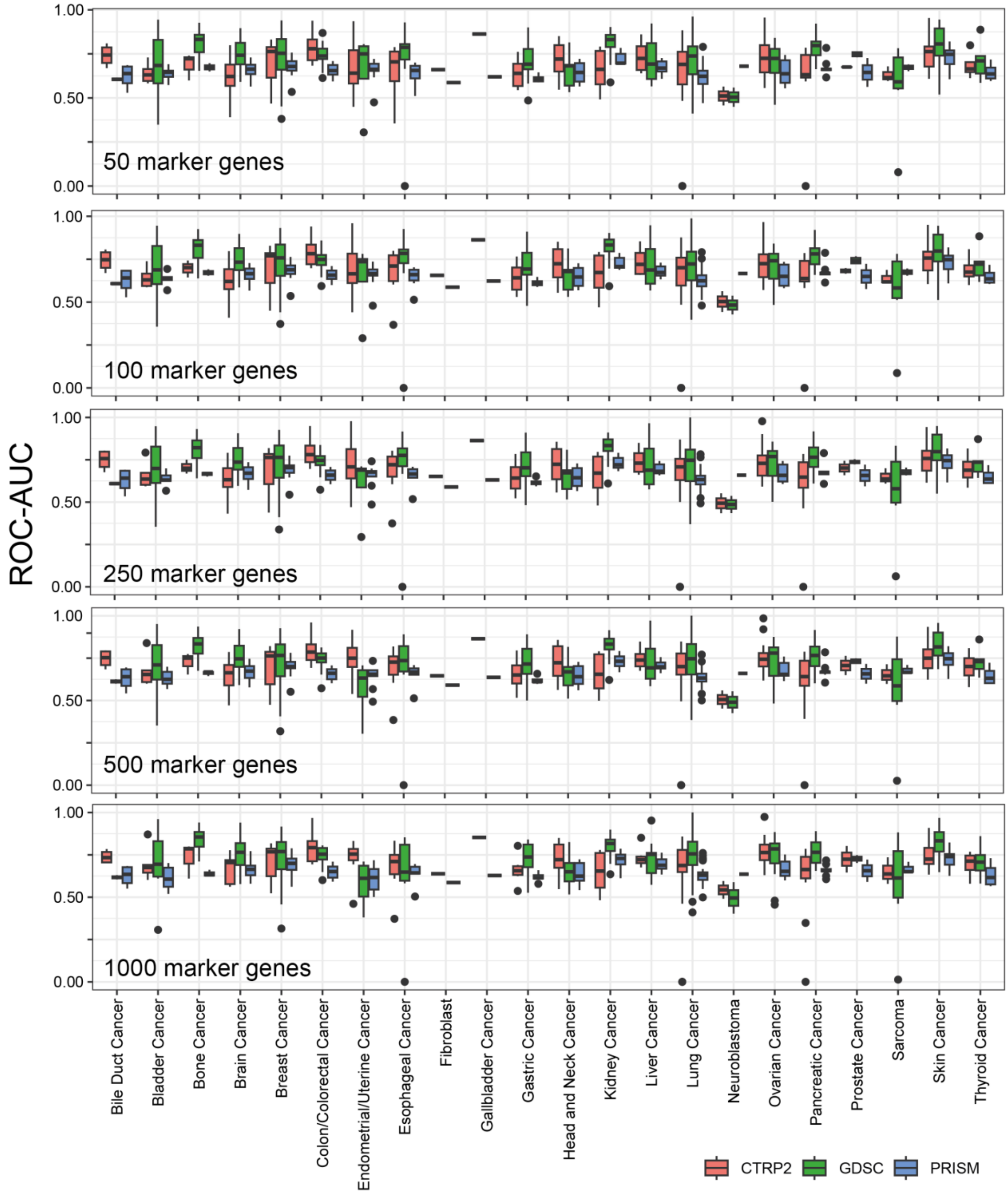

Figure S2 – DREEP's ROC AUC distribution across cancer types. This figure displays the ROC AUC distribution of DREEP across various cancer types, based on different sets of GDPS (CTRP2, GDSC, and PRISM). The distribution is presented concerning the number of relevant genes in the cells used to predict the effect of a drug.

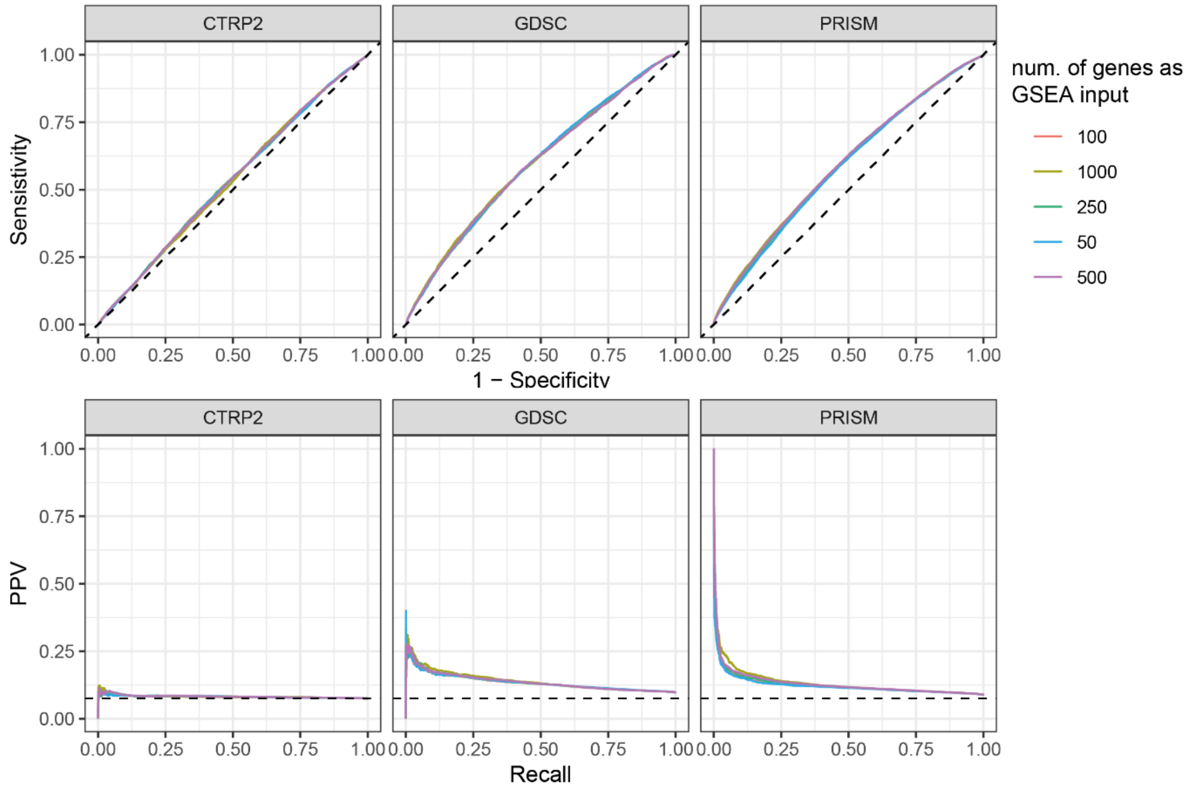

Figure S3 – DREEP Method Performance on Pan-Cancer Dataset using IC50-Derived GPDS. This figure assesses the performance of DREEP drug sensitivity across 198 cell lines, utilizing different sets of GPDS signatures and varying numbers of relevant genes for drug effect prediction. DREEP's performance is evaluated through Precision-Recall curves (bottom) and ROC curves (upper). Notably, the GPDS in this analysis have been constructed using drug IC50 values instead of their dose response AUC values.

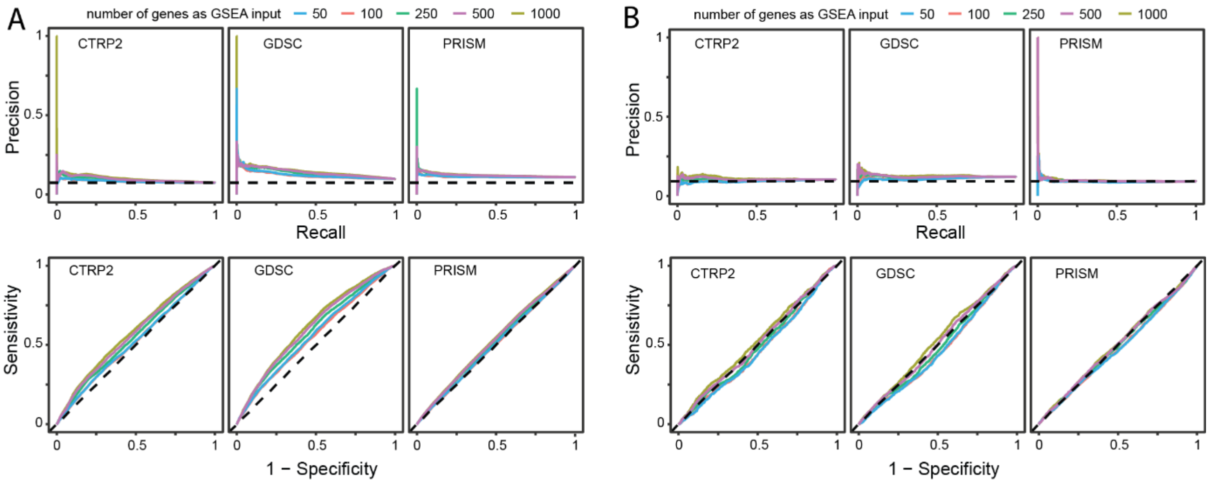

Figure S4 – Drug prediction performance using absolute expression to extract relevant genes of a cell. (**A**) Performance of DREEP drug sensitivity on 198 cell lines for each set of GPDS signatures used and different numbers of most expressed genes to predict the effect of a drug. DREEP's performance is estimated using either Precision-recall curve (upper) or ROC curve (bottom). (**B**) Same as (A) but for the 32 breast cancer cell lines published in Gambardella et al.

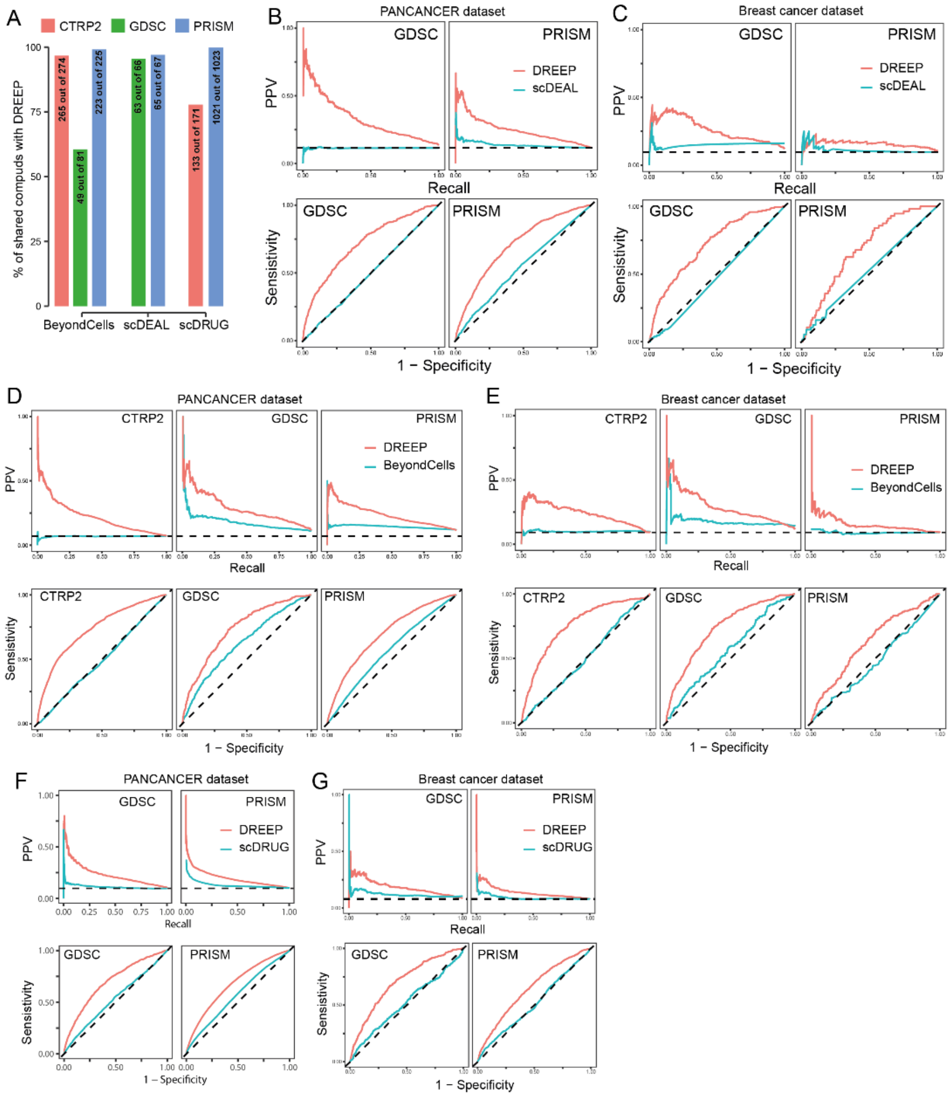

Figure S5 – Performance comparison between DREEP and other single cell drug prediction tool. Comparative Performance Analysis of DREEP and Other Single Cell Drug Prediction Tools. (**A**) Evaluation of shared drugs between DREEP and respective methods indicated on the x-axis across the three viability datasets: GSCS, CTRP2, and PRISM. Performance comparisons were specifically conducted on these shared drugs. (**B-G**) ROC curves (upper) and Precision-Recall curves (bottom) are presented, with DREEP shown in red, and the corresponding method indicated in the legend depicted in blue, across various viability datasets.

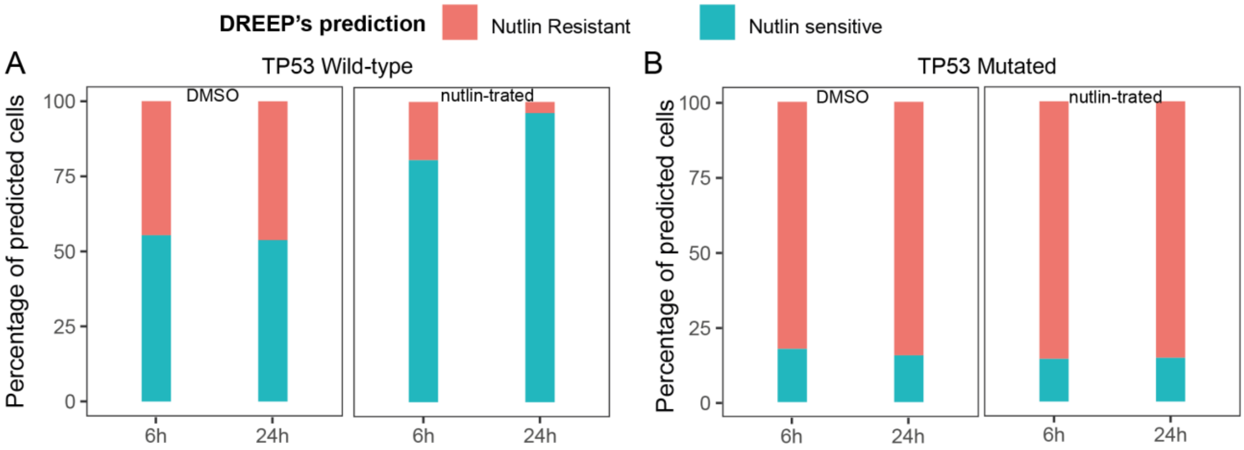

Figure S6 – DREEP's predictions for McFarland et al. Dataset. This figure illustrates DREEP's predicted percentage of sensitive and resistant cells to nutlin on both nutlin-perturbed and untreated (DMSO) cells. The cell populations are further stratified into TP53 wild-type (**A**) and TP53 mutated groups (**B**) for a comprehensive analysis.
